# SUPPORTING AGING IN PLACE WITH MOBILITY IMPAIRMENTS THROUGH ANNOTATED TELEPRESENCE TECHNOLOGY

**DOI:** 10.1093/geroni/igac059.2767

**Published:** 2022-12-20

**Authors:** Ben Thompson, Maribeth Gandy, Kala Jordan, Laura Levy

**Affiliations:** Georgia Institute of Technology, Atlanta, Georgia, United States; Georgia Institute of Technology, Atlanta, Georgia, United States; Georgia Institute of Technology, Atlanta, Georgia, United States; Georgia Institute of Technology, Atlanta, Georgia, United States

## Abstract

Most smarthome and smartphone technologies are designed with younger adults in mind, even though many older adults can benefit greatly from their use. Through our examination of how to make these technologies more accessible to adults aging in place with mobility impairments, we have found remote troubleshooting to be a possible solution to many problems. However, we have found most current teleconferencing solutions to be lacking in the limited perspectives of a single webcam, as well as from a lack of ability to point out specific items and locations within a camera view. As such, Our team developed a prototype system that used multiple desk space and room space views, as well as on screen annotations, to facilitate remote troubleshooting and social interactions through a teleconferencing service. This prototype was then evaluated with adults aging in place with mobility impairments, as well as accessibility experts, in order to see what parts of the system could be utilized for troubleshooting, as well as to see where the system was lacking. The studies tested the system’s ability to be used for troubleshooting, as well as their uses in games and social interactions. We have gathered positive feedback for these prototypes in the areas of troubleshooting, socialization, games, and for use in other accessibility studies. From these results, we have begun development of a mobile deployment kit for use of our teleconferencing solution in other accessibility studies.

